# Current challenges and the way forwards for regulatory databases of artificial intelligence as a medical device

**DOI:** 10.1038/s41746-026-02407-w

**Published:** 2026-02-11

**Authors:** Ariel Yuhan Ong, Aditya U. Kale, Joe Antoun, Henry David Jeffry Hogg, Ben Hammond, Pearse A. Keane, Russell Pearson, Hugh Harvey, Alastair K. Denniston

**Affiliations:** 1 Moorfields Eye Hospital NHS Foundation Trust, London, United Kingdom; 2 Institute of Ophthalmology, University College London, London, United Kingdom; 3 NIHR Moorfields Biomedical Research Centre, London, United Kingdom; 4 Oxford Eye Hospital, Oxford University Hospitals NHS Foundation Trust, Oxford, United Kingdom; 5 University Hospitals Birmingham NHS Foundation Trust, Birmingham, United Kingdom; 6 College of Medicine and Health, University of Birmingham, Birmingham, United Kingdom; 7 NIHR Birmingham Biomedical Research Centre, Birmingham, United Kingdom; 8 Hardian Health, Haywards Heath, United Kingdom; 9 Medicines and Healthcare Products Regulatory Agency, London, United Kingdom; 10 Birmingham Health Partners Centre for Regulatory Science and Innovation, Birmingham, United Kingdom

**Keywords:** Business and industry, Computational biology and bioinformatics, Health care, Mathematics and computing, Medical research, Scientific community

## Abstract

Effective regulatory oversight is a key step in ensuring that artificial intelligence as a medical device (AIaMD) is safe in real-world clinical settings. In this Perspective, we provide insights from our experience working with international regulatory databases, informed by our recent research and the expertise of the multidisciplinary authorship team. We highlight four key challenges, discuss attempts to circumvent these limitations, and highlight emerging initiatives. Nevertheless, the underlying issue of the quality and availability of input data from regulatory databases remains. We discuss considerations for improving accessibility and transparency, outline key aspects for a next-generation regulatory data ecosystem for AIaMDs, and call on global stakeholders to come together and align efforts to develop a clear roadmap to accelerate safe innovation and improve outcomes for patients worldwide.

## Introduction

Artificial intelligence (AI) has the potential to transform healthcare. While evidence suggests that AI can approach or even surpass human performance^1,2^, and potentially even develop unexpected or emergent capabilities^3^, it is crucial to ensure that the promising in silico performance translates to improvements in clinical practice whilst ensuring that patient safety is prioritised. Robust regulatory oversight is an essential factor in ensuring that AI as a Medical Device (AIaMD) - software incorporating AI intended for a medical purpose and regulated under existing medical device frameworks, also termed AI/machine learning (ML)-enabled medical devices in certain jurisdictions - work safely for patients and the public.

Alongside new opportunities, AI health technologies bring new risks which can challenge current approaches to medical device regulation. These include the risk of bias and variable performance across patient subgroups; risk of poor generalisability from one setting to another; and risks associated with a lack of interpretability of many models, which limits human verification and may negatively influence user confidence and trust^4^. In addition, frequent (and potentially continuous) updating of algorithms may lead to divergence from originally tested versions without the same level of oversight^4^. Regulatory systems around the world are trying to catch up with these challenges whilst also enabling safe and beneficial AIaMDs to enter the market. However, this remains a work in progress, and the extent to which current and emerging approaches address these risks is as yet unknown.

This evolving landscape underscores the importance of robust mechanisms for tracking and disseminating information about AIaMDs, including their regulatory approvals, clinical evidence, and safety profiles. Regulatory databases should aim to provide transparent and accessible information to stakeholders for multiple reasons: to assist regulators with information about their market; to support more informed selection and usage of technologies by clinicians and procurement teams; to enable the provision of better information and accountability to patients and the public; to provide market intelligence that may inform product specialisation, set benchmarks and encourage better investment decisions for manufacturers; and to facilitate discovery and identification of trends (including cross-sector safety issues) by research communities.

Essentially, better information supports better decisions, and can lead to more efficient, safer adoption and usage of AIaMDs within health systems, with actual benefit to patients. However, existing regulatory databases are inadequate for these purposes.

In this Perspective piece, we discuss challenges that we (and others) have encountered in navigating existing regulatory databases, the search strategies that have been attempted to circumvent current limitations in these databases, as well as the role of AI registries and new initiatives in bridging this information gap. Building on these insights, we propose a conceptual model which highlights considerations for developing more transparent and interoperable regulatory databases, and outline a roadmap from fragmented and isolated national systems towards a global learning data ecosystem that sustains continuous post-market surveillance.

## Challenges in navigating regulatory databases

The limitations of current regulatory databases were highlighted during the delivery of two recent reviews by our research group, which relied on medical device databases across the European Union (EU), Australia, and the United States of America (USA). The first was a systematic review of adverse events related to AIaMDs which were reported in the regulatory databases of those jurisdictions^5^. The second was a scoping review of AIaMDs approved for ophthalmic image analysis (e.g. screening for diabetic retinopathy) in those same jurisdictions^6^. To put our findings in a more global context, we have also reviewed a sample of databases hosted by regulatory agencies in other jurisdictions, which suggest that these challenges are common to the databases provided by leading regulators across the world. We present the results of these searches in Table 1, with the caveat that this table represents a cross-sectional overview of 20 key regulatory databases (including members of the International Medical Device Regulators Forum and others) which is accurate as of May 2025.

**Table 1:** Summary of medical device databases from a sample of regulatory bodies in different jurisdictions

Country/ region	Regulatory body	Medical device database	Public access	Database transparency			Comments^a^
				Search functionality	Safety/Adverse events	Clinical validation	
Australia	Therapeutic Goods Administration (TGA)	Australian Register of Therapeutic Goods (ARTG)	Yes	Limited: product name or ID, sponsor name, manufacturer name. Free-text search functionality.	Database of Adverse Events Notification (DAEN), searchable by device type. Safety Alerts, searchable listings	No	Brief public-facing summary including intended purpose
Brazil	Agência Nacional de Vigilância Sanitária – Anvisa (ANVISA)	Unnamed database	Yes	Limited: queries based on device name or registration, sponsor name or registration	Technovigilance Alerts: provides alert reports across multiple industries (including food, aviation, healthcare etc.	ANVISA Clinical Investigation Database	In Portuguese, English translation available
Canada	Health Canada	Medical Devices Active Licence Listing (MDALL)	Yes	Limited: queries based on device name and identifier; company name, ID, license name and number	Canada Vigilance Adverse Reaction Online Database	No	
China	National Medical Products Administration (NMPA)	NMPA Medical Device Database (in Mandarin Chinese) *link not working as of May 2025 List of announcements (available in English)	Yes	Limited: queries based on product code, device name, company name	National Medical Device Adverse Event Monitoring Information System - not publicly available, annual report published up to 2020	No	Information in Chinese
European Union	European Commission	EUDAMED (in development, delayed)	Yes	Limited: queries based on product code, device name/ class/ type, company name, medical purpose	Not yet, but vigilance and post-market surveillance modules planned. Currently, each EU jurisdiction maintains their own safety/ adverse event database	No, but clinical investigation database planned	EUDAMED remains in development with delays to transition period
Ireland	HPRA	No publicly available database	No	Unknown	List of field safety notices and monthly summary	No	Northern Ireland covered by PARD (UK); republic of Ireland by EUDAMED
Switzerland	Swissmedic	swissdamed (swiss database on medical devices)	Yes	Very limited: queries based on manufacturer name and location; device name and UDI planned for future iterations	List of field safety notices available on swissdamed	No	
France	Agence nationale de sécurité du médicament et des produits de santé (ANSM)	Unknown	No	Unknown	List of product recall notices and safety information filterable by type and date	No	Information in French only
Germany	Bundesinstitut für Arzneimittel und Medizinprodukte, (BfArM)	German Medical Devices Information and Database System (DMIDS)	Partially publicly accessible (Medical Devices Notification only)	Very limited: only information on medical device notification and company address is available publicly	List of Field Corrective Actions	No	Information in German only
India	Central Drugs Standard Control Organisation (CDSCO)	Online System for Medical Devices *link not working as of May 2025	Yes	Limited: keyword searches for manufacturer/ importer name and license; device name, class, model number; issuing authority	Materiovigilance Programme of India (MvPI) – not publicly accessible	No	
Japan	Pharmaceuticals and Medical Devices Agency (PMDA)	PMDA Medical Devices	Yes	None: lists approved devices; provides annual summaries	MHLW Pharmaceuticals and Medical Devices Safety Information (list)	Yes – provides review of submitted data	Information in Japanese with English translation
Malaysia	Medical Device Authority (MDA) Malaysia	Medical Device Authority Register (MDAR)	Yes	Limited: product name, registration number; importer name	List of field safety notices and product recalls	No	
New Zealand	Medsafe (the New Zealand Medicines and Medical Devices Safety Authority)	Web Assisted Notification of Devices (WAND) Database	No – restricted to sponsors	Not publicly available	No adverse event database but has a recalls database (Medsafe Online Recalls Database) searchable by brand name and date	Unknown	
Russia	Russian Ministry of Health	Not publicly available	Not publicly available	Not publicly available	Not publicly available	Unknown	Information in Russian
Singapore	Health Sciences Authority (HSA)	Singapore Medical Device Register (SMDR) Class A Medical Device Database	Yes	Queries based on product name, identifier, class; manufacturer name, registration, importer; country; medical specialty	HSA Adverse Event Online Database - access limited to manufacturers and healthcare professionals	No	
South Africa	South African Health Products Regulatory Authority (SAHPRA)	SAHPRA Medical Devices License Database - details on manufacturers with medical device licenses only	Yes but provides limited information	Very limited: keyword searches for company name, license type and number, address, issue data	No publicly available database	No	
South Korea	Ministry of Food and Drug Safety (MFDS)	Annual reports aggregate approvals, certifications, and notifications of medical devices MFDS Medical Device Lifecycle Research Database	Yes	Unknown (NB: MFDS database mentioned on multiple sites, but database itself cannot be located)	Unknown	Unknown	Information in Korean
United Kingdom (UK)	Medicines and Healthcare products regulatory agency (MHRA)	Public Assessment Registration Database (PARD)	Yes	Limited: queries based on device type and manufacturer name only	Separate database hosted on gov.uk listing alerts, notices, and field safety notices; limited search functionality	No	Post-Brexit regulatory reforms are in progress
United States of America (USA)	Food and Drug Administration (FDA)	FDA Medical Device Databases	Yes	Limited: queries based on device name, class, product code, submission type etc.	Manufacturer and User Facility Experience (MAUDE) for adverse events Medical Device Recalls Database	Yes	

Note 1: This information was extracted by one author and verified by a second to optimise accuracy. However, regulatory databases may be subject to updates or structural changes over time; this table therefore represents a cross-sectional overview that is accurate as of May 2025.

Note 2: ‘Unknown’ means that the information could not be verified because there was insufficient information in the public domain to determine whether the database exists. ‘Not publicly available’ means that the database exists, but is not publicly available.

^a^All databases were available in English as a primary language unless specified otherwise.

The four key challenges can be summarised as follows:

1. Limited search functionality: When attempting to compile a list of relevant AIaMDs, we found that most regulatory databases lacked intuitive and consolidated search capabilities, such as a comprehensive keyword-based search functionality. Although several - such as the FDA (USA), EUDAMED (EU), and HSA (Singapore) - had ‘advanced’ search options, these were often limited and challenging to navigate, particularly for non-experts, who may not have access to specific information such as product code, registration number, or other input fields which may be difficult to interpret without highly technical knowledge. In addition, product name or manufacturer name may differ in each jurisdiction, adding to the complexity of the search.
2. No specific terminology for identification of AIaMD: Tracking regulatory approvals and adverse events specifically related to AIaMDs was challenging because most databases did not specifically tag medical devices as being AI-enabled. This was mainly because AI-specific terms have not been included in key nomenclature, such as the Global Medical Device Nomenclature (GMDN) used by several regulators, or the European Medical Device Nomenclature (EMDN) used in the EU. The FDA (USA) deals with this by maintaining a separate manually curated list of AI/ML-enabled medical devices^7^. However, the definition or threshold for ‘AI/ML-enabled’ is unclear, and our analysis of supporting information for some of these devices (undertaken during our reviews) cast some doubt on whether they had been appropriately classified. Others have reported similar issues in examining official FDA announcements of regulatory approvals^8^. In many jurisdictions, the onus is on the manufacturer to report whether their device is AI-enabled, meaning that these claims may be at risk of being influenced by investment and marketing considerations, rather than being based on the technology itself.
3. Limited transparency: Several regulatory bodies do not maintain publicly accessible databases, and some restrict access to specific entities or only make limited parts of the database available. Among those with public-facing platforms, the majority provide basic device-level information such as the product name, manufacturer, and class in tabular format, with some such as PARD (UK) not disclosing device names at all. Very few agencies, such as the FDA (USA) and PMDA (Japan), publish information on clinical evaluation studies submitted for regulatory approval, but the level of detail varies across submissions. Overall, there is a lack of prospective trials (especially those reporting safety and real-world performance) for AI and digital health technologies in the pre-market phase^9^, which limits meaningful evaluation of risks and benefits at the point of approval. Greater transparency in regulatory databases, including consistent publication of trial protocols and outcomes, will be helpful in addressing this gap. While EUDAMED (EU) is expected to mandate the disclosure of clinical investigation data in the coming years, the granularity of this data is currently very limited^10^.
4. Data fragmentation across multiple databases: Details on regulatory approvals, post-market surveillance, adverse events, and product recalls are typically stored in separate databases, each with its own data structure, search capability, and nomenclature systems. This siloed approach necessitates the consultation of multiple sources to reconcile conflicting or incomplete information. As a result, tracking AIaMD performance across their entire lifecycle - from pre-market evaluation to real-world deployment and post-market surveillance - becomes more complex, and identifying critical safety signals or emerging performance concerns becomes even more challenging. The EUDAMED (EU) database is moving towards greater integration by mandating and linking multiple modules (including device and actor registration, clinical investigation, market surveillance, and post-market surveillance), with full implementation planned in the next few years^10^.

## Search strategies that address current database limitations

In summary, current iterations of regulatory databases all have significant limitations, and are highly variable in the data they provide and the format they provide it in. The following reflects the approaches that we and other researchers have taken when trying to search these databases for AIaMDs:

### Identifying AIaMDs

To address the difficulties in identifying eligible AIaMDs, we applied a “snowball” search strategy to all regulatory databases, starting with a list of AIaMDs from those known to us and from a pragmatic search of the relevant literature. We then performed an exhaustive review of the product class codes and predicate devices (where applicable) with which each known eligible device was associated in each database, and then screened the resulting list of devices with similar product class codes^6^.

Other researchers have developed creative strategies to circumvent the limited search functionality of existing regulatory databases and the lack of AI-specific nomenclatures (GMDN and others). Examples include applying automated programmes, natural language processing (NLP) techniques, or targeted keyword searches to regulatory databases; searching online sources (including manufacturer websites, news aggregation websites, news sources, product advertisements) using similar techniques, with or without cross-validation against a regulatory database; or a combination of the above^9,11–14^.

### Clinical evaluation

Since most regulatory databases provide no or very limited clinical evaluation data, our approach was to supplement this by searching biomedical literature databases (e.g. PubMed), hand-searching references of relevant papers, reviewing manufacturer websites, and contacting manufacturers^6^, similar to approaches taken by other groups^14^. This approach has significant limitations, since it cannot overcome the fundamental lack of transparency of the current system. Manufacturers have no obligation to publish the evidence they provide to regulators and may claim that this is commercially sensitive. Whilst they may choose to publish in academic journals or other publicly available repositories - modelling openness and potentially raising their profile - many manufacturers do not do this^6^. In some cases, partial publication of data may occur, for example through conference abstracts, which often have less stringent requirements and a lower bar of peer review. Open and transparent reporting of clinical evaluation data should be strongly encouraged, and ideally mandated. It would seem to be a reasonable expectation on behalf of a health system that pays for a device, for practitioners who use the device, and for patients whose care depends on a device, that the manufacturer would provide all available evidence regarding performance and safety, and that ongoing performance and safety data should be efficiently and rapidly shared across jurisdictions and stakeholders.

Another challenge in identifying relevant clinical evaluation data is in situations where there has been a change in device name, either between jurisdictions or between time periods, for example between publication of a clinical study and market authorisation^6^. Public-facing materials such as company websites may not always provide sufficient detail, especially for identifying earlier versions of the product. Even when the lineage of a device has been correctly identified and the clinical evidence collated, it may also be challenging to ascertain how relevant clinical data from an earlier version of a model is to the latest model on the market. Recognising the limitations of public-facing documents, our approach was always to contact the manufacturer, but response rates can be highly variable. It is a limitation of the current system that openness and transparency is highly dependent on voluntary information sharing by AIaMD manufacturers^6^.

### Adverse events and safety

To identify and analyse adverse events, we searched regulatory databases containing adverse event reports and field safety notices. This was highly burdensome, since search strategies had to be adapted for each database to account for discrepancies in terminology, device names, and database search functionality. The quantity, quality, and depth of information provided varied by device, manufacturer, and regulator, necessitating a combination of manual screening for eligibility (due to medical device nomenclature lacking AI-specific codes) and pragmatic sampling to analyse the large volumes of data. Where feasible, automated scripts were employed to systematically collect and collate results. NLP techniques were attempted but ultimately fell short of the required accuracy due to the level of complexity. Availability and amount of detail for adverse event reports varied not only across jurisdictions, but also between reports. This is a known issue owing to the largely voluntary nature of adverse event reporting. However, under FDA guidance, adverse event reporting is mandatory for reporters such as manufacturers, and is optional for healthcare professionals, patients, and carers, who are encouraged to do so^15^. This will soon become mandatory for manufacturers in the EU under the EU MDR, after the EUDAMED vigilance module is deployed^16^.

Even with highly targeted regulator-specific approaches to these searches, the quality and completeness of the data available was very variable. Again, this is a serious limitation of the current system, since it is difficult to get a clear picture of the safety and performance profiles of AIaMDs even with a high level of effort and reviewer expertise. The data gleaned from these approaches comprises different data fields, reported in different ways across multiple jurisdictions. It provides a hazy view, like driving a car in severe fog: we can see enough not to hit a truck, but not enough to spot less obvious objects. As noted for medical devices generally - and specifically for AIaMDs here - there is an urgent need for more systematic, harmonized and transparent reporting practices relating to their performance and safety.

## The role of AI registries and new initiatives

In the absence of adequately accessible regulatory databases that provide the information that users need, others - predominantly speciality-specific professional bodies or commercial entities - have sought to bridge this information gap.

Professional bodies such as the American College of Radiologists (ACR) in the USA, and the Royal College of Radiologists (RCR) and Royal College of Ophthalmologists (RCOphth) in the UK, have developed registries for AIaMDs focused on their respective specialties and jurisdictions. The ACR platforms include AI Central, a searchable directory of FDA-authorised AIaMDs with radiology applications, as well as the newly launched ASSESS-AI, which aims to capture real-world post-deployment data to enable performance monitoring^17^. The RCR AI Registry is a searchable database focusing on AI-enabled radiology products, which highlights basic details about the product and information on deployment around the UK^18^. The RCOphth AI Directory performs an analogous function for ophthalmic AIaMDs, albeit with a more restricted range and lack of search function given the less mature field^19^.

The offerings from commercial entities tend to be broader, either in terms of geographical reach or functionality, although this also depends on the extent of manual versus automated curation. The Health AI Register from Romion Health is limited to AI-enabled radiology products, but covers both the EU and USA. It provides a manually curated overview of the product characteristics, technical specifications, regulatory approval details, and supporting clinical evidence where available^14,20^. As the name implies, BEUDAMED (Better EUDAMED) from OpenRegulatory aims to improve the user experience and search functionality of EUDAMED (EU) by simplifying the search interface from multiple specific input fields to a single text field, enabling filtered searches, providing search suggestions, and speeding up searches^21^. However, this does not improve the quality or utility of the underlying data. A product that seeks to extend functionality and breadth of coverage is HaRi (Hardian Regulatory Intelligence) from Hardian Health. Currently in beta testing, HaRi is a platform which aggregates salient information from multiple regulatory databases^22^. At the time of writing, it covers EUDAMED (EU), MHRA (UK), Health Canada (Canada), and FDA (USA), with additional features including integration with PubMed to highlight relevant clinical studies, as well as adverse events and device recall databases to address safety concerns.

Each approach has advantages and disadvantages. Manual curation enables quality control by human experts, but the volume of data renders expanding beyond specialty-specific solutions unfeasible. Automated data aggregation or scraping of regulatory databases may require careful oversight to maintain the precision and relevance that manual curation offers. Both depend on the quality of the input data, which will be limited without publicly accessible, transparent, and well-resourced regulatory databases. However, this may not be a priority for regulatory bodies that face competing demands for their limited time and resources (and in some cases major disinvestment). The result is a fragmented landscape where overlapping efforts may lead to inefficiencies that could be avoided with a more unified and collaborative approach.

## Considerations for publicly accessible and transparent regulatory databases

Importantly, these concerns are not unique to AIaMDs alone. Fragmentation, opacity, and delays in regulatory responsiveness are longstanding systemic issues in medical device regulation. High profile failures, such as the delayed regulatory response to the PIP breast implant scandal, have drawn attention to this globally^23^. In response to this, the Cumberlege review in the UK has highlighted serious limitations in post-market surveillance and adverse event reporting systems, which has prompted recent legislative changes^24,25^. However, there remains a clear need for robust, transparent, and accessible regulatory infrastructure that spans the full medical device lifecycle, including interoperable public-facing databases to facilitate monitoring, accountability, and evidence generation across all classes of medical devices. Whilst important for medical devices generally, this is perhaps especially important for AIaMDs, given some of their unique characteristics discussed earlier, including risks of poor generalisability, risk of bias, and manufacturers’ expectations of frequent updating^4^.

These examples also raise the critical question of governance: who should be responsible for developing, overseeing, or maintaining these databases or registries? Should the onus be on regulatory bodies alone to improve their database infrastructure, or should non-governmental agencies, non-profit organisations, or inter-governmental organisations administer separate registries that may or may not be built on these regulatory databases? And how do commercial entities and professional bodies factor into this equation?

In addition, at which stage in the AIaMD life cycle should it be registered, and what data should be collected? While current regulatory databases only include market-approved devices, some investigators have argued that even early-stage algorithms should be registered^26^. The extent of data collected and provided could range from up to market approval only (relatively easy for regulators), to the provision of various levels of post-market performance and safety data, the most extreme of which would be complete, standardised near-time reporting of all AIaMDs in deployment^27^, extending from their local validation pre-implementation to ongoing monitoring as part of local clinical safety, quality assurance and post-market surveillance^28^. Despite post-market surveillance being mandatory, a robust strategy for post-market surveillance of AIaMDs remains to be determined, partly due to recognised logistical constraints on getting information back from digitally fragile health systems. Again, this is not specific to AIaMDs. Even for the broader category of medical devices, this remains insufficiently reproducible, evidence-based, or internationally harmonized^29^.

## Toward a federated and learning regulatory data ecosystem

**Fig. 1:**
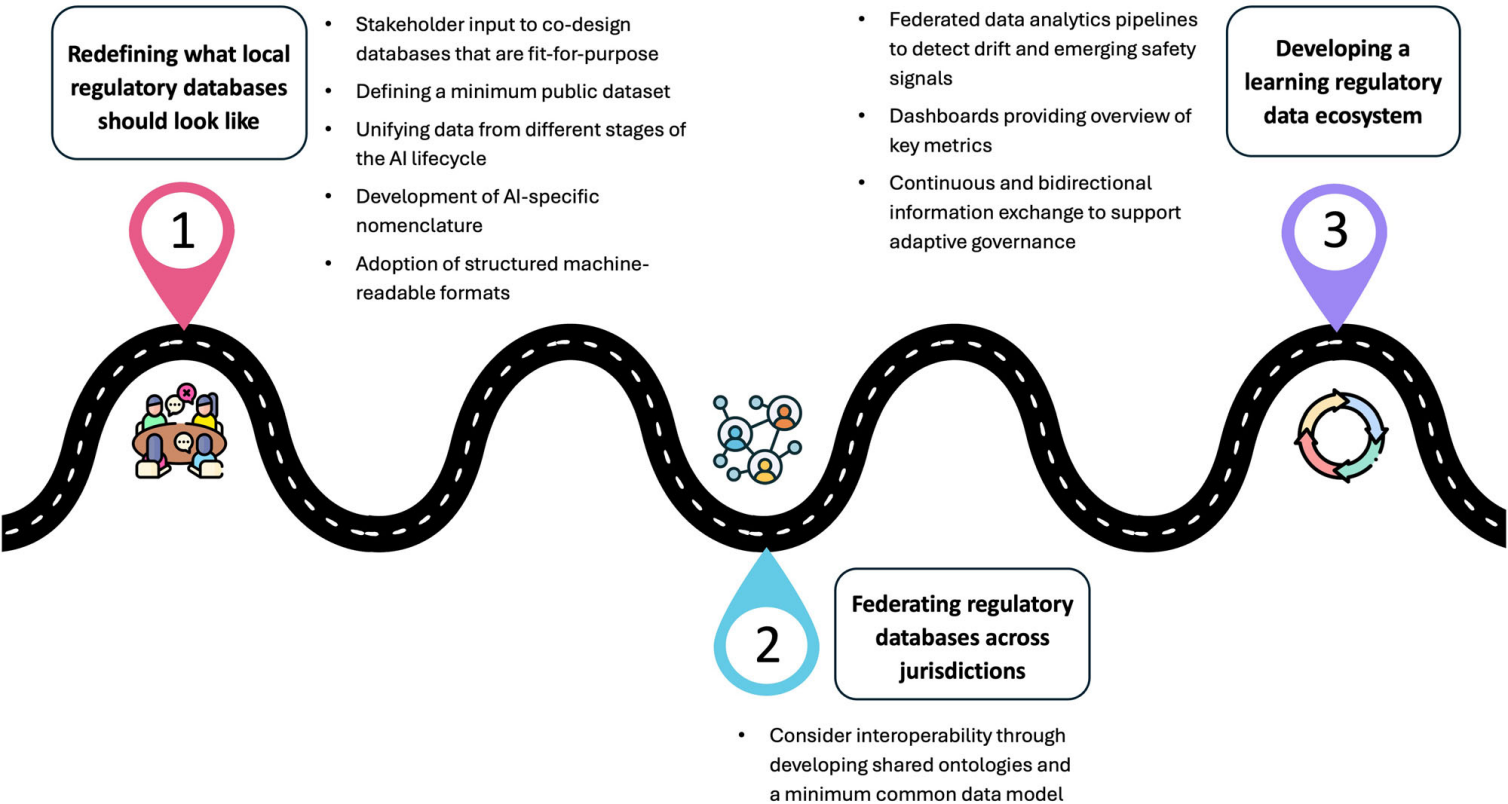
One potential path towards a next-generation regulatory data ecosystem for artificial intelligence as a medical device (AIaMD). Phase 1 focuses on co-designing local databases that meet stakeholder needs. Phase 2 links these local regulatory databases through federated infrastructure to enable cross-jurisdiction oversight and harmonised analytics. Phase 3 transforms the federated network into a learning regulatory ecosystem, where real-world evidence informs continuous, adaptive regulatory decision-making, supporting safer and more effective AIaMD approval and deployment.

Ultimately, effective oversight of AIaMDs depends on the quality of the data that underpin them. Evaluating these technologies across their full lifecycle, from regulatory approval to real-world performance, requires modern regulatory databases that support traceability, interoperability, and adaptive oversight. Building on this, we propose one possible path towards a next-generation regulatory data ecosystem that maintains jurisdictional sovereignty by design (Fig. 1).

### Phase 1: Building regulatory databases that are fit for purpose

While the first step is to build regulatory databases that meet stakeholder needs, the lack of functionality belies deeper issues. These include fragmented data infrastructure with incompatible data schemas, static reporting formats, and variable linkage to device versions or updates. Overcoming these issues requires a fundamental rethinking of how the regulatory data ecosystem is designed and governed.

This matters because regulatory databases are not simply static repositories of device approvals, but also tools for traceability and learning across the medical device lifecycle. For manufacturers of medical devices (including AIaMDs), access to detailed data on the market and existing regulatory approvals can support more targeted development and a faster path to market opportunities, including greater clarity regarding regulatory expectations^30^. For purchasers, practitioners and patients, detailed performance and safety data (including up-to-date indicators of safety signals for rare events) are the basis of sensible procurement and safe use of such technologies. This can also help facilitate and standardise procurement decisions for healthcare commissioners and other key decision-makers, providing key evidence for them to perform their due diligence.

As such, improvements to regulatory databases might involve unifying different stages of the AI lifecycle – from regulatory approval, to clinical evaluation, to post-market surveillance and beyond. Although not all regulatory and post-market data can be shared for legal, commercial, and ethical reasons, a minimum public dataset could be published for every approved device, with metadata indicating withheld information where necessary. These need not threaten manufacturers’ need to protect commercially sensitive information with regard to how a technology works, whilst allowing them to remain open and transparent about its performance. Various reporting frameworks have set expectations regarding what should be published in relation to an AI health technology (including performance metrics, training and test datasets, and in some cases the details of the code)^31,32^. Transparent databases containing accessible clinical evaluation data, including post-market surveillance studies and adverse event reports, can reduce the risk of publication bias and provide a more complete picture of an AIaMD’s real-world safety and efficacy. This additional source of evidence can enable independent critical appraisal and validation of the claims made by manufacturers, facilitate evidence synthesis, and allow for comparative studies across different jurisdictions, such that patterns in regulatory decision-making processes, standards, and outcomes can be identified. When designed and governed in this way, they become living systems that simultaneously strengthen innovation and protect patients.

To this end, we propose that early engagement with these diverse stakeholder groups is essential to co-design databases that are fit for purpose. These includes defining the information required, how often it must be updated, and the level of technical detail necessary, and which parts should be public-facing. Basic principles of user design and user experience (e.g. intuitive search functionality) should also be considered, with stakeholder input. In addition, development of AI-specific nomenclature (building on existing nomenclature systems such as the GMDN) should be prioritised to enable appropriate identification and categorisation^13,33^. Databases could also adopt structured, machine readable formats with standard identifiers (e.g. unique device identifiers [UDI], which are already used by several regulatory bodies) to ensure that devices can be consistently tracked across jurisdictions, linked across versions, and integrated with other health and regulatory data sources.

### Phase 2: Federated analysis across regulatory databases

A natural next step might be to connect the strengthened local regulatory databases across jurisdictions. Previous authors have proposed a federated registration system for AIaMDs, which includes the development of local registries to record and track all AI systems deployed in clinical care and operations, followed by federation at a national level to improve traceability^27^. Extrapolating from this, we ask: beyond federating local clinical registries alone, what if we can federate regulatory databases themselves to create a globally connected infrastructure that spans the entire AIaMD lifecycle?

AIaMDs are often developed, approved, and updated across multiple jurisdictions, meaning that this approach could potentially reduce duplication of manufacturer submissions, promote consistency in evidence requirements, and enable comparative analyses of regulatory decisions. Federated infrastructure would allow regulators to share harmonised metadata and performance and safety information while maintaining sovereignty over sensitive data, thereby balancing transparency with data protection^34^. This alignment would enable regulators to detect early safety signals, monitor performance drift, and benchmark evaluation processes across jurisdictions, which would provide a more comprehensive view of real-world device performance as well as facilitating coordinated regulatory responses. Federated analysis can be achieved through secure application programming interfaces (APIs) that exchange standardised metadata (comprising identifiers, AI-specific descriptors, update history, and post-market data) without requiring central data pooling. Importantly, because participation is voluntary and controlled by individual regulatory bodies, each jurisdiction maintains sovereignty over their regulatory frameworks and databases. This proposal may therefore support regulators in reducing information asymmetries to mitigate regulatory arbitrage, should they choose to do so.

This proposal borrows from the concept of federated health data networks^34^, which also share similar interoperability challenges from heterogenous health data^35^. To address this, shared ontologies and a minimum common data model could be agreed through mechanisms such as the IMDRF. Parallels may also be drawn with the World Health Organisation’s International Clinical Trials Registry Platform, which provides a single point of access for clinical trials from 17 databases internationally, by first setting standards for trial registration^36^. While this example relates only to publicly accessible information and is aggregated rather than truly federated, it demonstrates the feasibility of harmonising decentralised databases through common standards and governance.

### Phase 3: Towards a learning regulatory data ecosystem

These foundations set the stage for transforming connected regulatory databases into a learning regulatory ecosystem. Rather than relying on periodic reporting cycles, post-market data (including post-market surveillance submissions and adverse event reports) can be analysed automatically through federated analytics pipelines that detect emerging safety signals and performance drift across populations and settings in near real time. The results could feed into shared dashboards that provide regulators, manufacturers, and health systems with an overview of key metrics such as approval timelines, recall frequency, and bias indicators. These insights could then inform updates to approval criteria, evidence standards, and post-market obligations to enable earlier intervention and safer iteration of AIaMDs. This continuous and bidirectional information flow moves oversight from static compliance to adaptive governance, and from reactive to proactive risk mitigation, embedding continuous improvement to ensure that AIaMDs remain safe, effective, and equitable throughout their lifecycle.

Ultimately, this is simply one possible vision. Realising it will require each regulatory body to have the analytic and computational capability to process large volumes of data securely without sharing the raw data; the legal and technical frameworks for data quality, versioning, and cross-border metadata exchange to ensure interoperability and accountability across jurisdictions; and a willingness to consider transparent public reporting and benchmarking, for example through aggregated dashboards to provide visibility into performance and safety, promote trust among clinicians and patients, and create incentives for continuous improvement. However, at present, regulatory incentives in many jurisdictions prioritise rapid innovation and market access, with comparatively weaker incentives for comprehensive lifecycle data generation and transparent post-market evidence. Progress would therefore require shifts in emphasis such as stronger incentives for ongoing real-world performance monitoring, clearer linkage between regulatory approval and monitoring, and sustained investment in regulatory infrastructure. These ambitions must be balanced against practical and logistical constraints including funding and staffing, stakeholder buy-in, as well as alignment of priorities across jurisdictions. Achieving this will require careful deliberations to build consensus, structured pilots to refine this vision iteratively, and the political and financial commitment to sustain long-term collaboration. In this sense, our proposed roadmap is best understood as a set of operational desiderata (desirable characteristics of a future regulatory data ecosystem) expressed as an evolutionary, phased pathway rather than an expectation of near-term realisation.

## Conclusion

The challenges surrounding regulatory databases for AIaMD reflect broader tensions in medical device oversight: fragmented databases, lack of transparency, and limited mechanisms for continuous oversight. The issues raised herein may be addressed by creating a collaborative model where industry, regulatory bodies, health systems and others set a shared expectation around information sharing, with the overall aim of streamlining processes and reducing duplication. Building on empirical observations and normative considerations, we outline one proposal for a path toward a federated and learning regulatory data ecosystem that integrates standardisation, shared governance, and transparency as core design principles. Could this even lead to a global AI registry, which would be open access and could enable the concentration of finite resources on improving data quality, transparency, and accessibility of existing databases? While this may be challenging to achieve given competing interests and resource availability, the current systems are inefficient and inadequate. No single sector or country can solve this alone. Achieving this vision will require sustained policy commitment, regulatory capacity building, and shared investment and buy-in across jurisdictions. But if we can get this right, the opportunity is clear – we can accelerate innovation in the AI and digital health space, improve health system efficiency, and ensure that patients can benefit from technologies with comprehensive, near-time safety data from around the world.

## Data Availability

All data generated or analysed during this study are included in this published article.
